# Human infection with avian influenza A(H7N2) virus—Virginia, 2002

**DOI:** 10.1111/irv.12546

**Published:** 2018-03-13

**Authors:** Pauline Terebuh, Akini Adija, Lindsay Edwards, Thomas Rowe, Suzanne Jenkins, Jennifer Kleene, Keiji Fukuda, Jacqueline M. Katz, Carolyn B. Bridges

**Affiliations:** ^1^ Centers for Disease Control and Prevention Atlanta GA USA; ^2^ Virginia Department of Health Richmond VA USA

**Keywords:** avian influenza, H7N2, human infection, low pathogenic, serology

## Abstract

**Background:**

In March 2002, an outbreak of low‐pathogenic avian influenza (LPAI) A(H7N2) was detected among commercial poultry operations in Virginia.

**Methods:**

We performed a serosurvey of 80 government workers involved in efforts to control the outbreak.

**Results:**

One study participant who assisted with disposal of infected birds tested positive for neutralizing antibodies to influenza A(H7N2) by microneutralization assay and H7‐specific IgM antibodies by enzyme‐linked immunosorbent assay (ELISA). The acute infection was temporally associated with an influenza‐like illness that resolved without hospitalization.

**Conclusion:**

This study documents the earliest evidence of human infection with an H7 influenza virus of the North American lineage.

## 1 INTRODUCTION

In March 2002, an outbreak of low‐pathogenic avian influenza (LPAI) A(H7N2) was detected among commercial poultry operations in Virginia. The Virginia Avian Influenza Task Force (VAITF) composed of government workers was formed to detect and control the outbreak that lasted 4 months, infected 197 turkey and chicken flocks, and resulted in the depopulation of 4.7 million birds.^1^ North American LPAI H7N2 viruses circulated in live bird markets (LBM) of the northeastern United States from 1994 to 2006 and have been a source of infection for this and multiple commercial poultry outbreaks.^2^, ^3^, ^4^ No human infections with LPAI H7N2 viruses had been documented prior to the 2002 Virginia outbreak. We describe a serosurvey conducted among persons involved in control efforts that resulted in identification of the first human LPAI H7N2 infection.

## 2 METHODS

We performed a serosurvey among eligible workers (≥18 years of age who had participated in control efforts of the LPAI H7N2 poultry outbreak in Virginia for at least 14 days by May 17, 2002) and who gave informed consent to enroll in the study. Outbreak‐associated and previous exposure to wild birds and poultry, the use of personal protective equipment during control efforts, and recent ocular and respiratory symptoms were assessed. A blood sample was collected from each participant for serologic testing. A second blood sample was collected from participants whose first sample tested positive for antibody to influenza A(H7N2). The study was approved by the CDC's institutional review board.

A LPAI A(H7N2) virus, A/Tky/VA/4529/2002 (Tky/VA), was isolated from the respiratory tract of a turkey from the index farm in the poultry outbreak and was used for serologic testing of human serum samples. Each serum sample was tested at least twice in separate microneutralization (MN) assays for the presence of neutralizing antibodies to Tky/VA virus using a starting dilution of 1:20 as described previously.^5^ Work with live Tky/VA virus was conducted in a biosafety level 3 laboratory with enhancements required by the US Department of Agriculture. Serum samples with duplicate titers of ≥80 were tested further by immunoblot and an enzyme‐linked immunosorbent assay (ELISA) detecting IgM also described previously,^5^ but using hemagglutinin protein derived from whole purified Tky/VA virus by treatment with bromelain.^6^ Additional testing was performed using adsorbed serum samples to rule out the possibility of antibody cross‐reactivity between H7 and the A(H3N2) subtype. A contemporary human A(H3N2) virus was chosen for the adsorption protocol because the individual exhibited substantial titers to A(H3N2) virus (data not shown). In addition, H3 and H7 HA are both phylogenetic group 2 HA subtypes, raising the concern that cross‐reactive antibody to the H3 HA could possibly interfere with the detection of authentic H7 subtype‐specific antibodies.^7^ Briefly, a 50 μL volume of serum was incubated with 100 μg of whole purified Tky/VA or purified A/Sydney/5/97 (H3N2) (Syd/97) viruses for 2.5 hours at 4°C followed by ultracentrifugation at 100 000 *g* for 30 minutes to remove virus‐antibody complexes. Viral protein was measured by Bradford assay (Bio‐Rad Laboratories, Hercules, CA, USA). To remove residual virus, sera were then adsorbed for 30 minutes with a one‐tenth volume of packed turkey red blood cells, followed by centrifugation at 21 000 *g* for 2 minutes at 4°C. Absorbed serum samples were tested by MN assay and ELISA using Tky/VA H7 HA and A/Panama/2007/99 (H3N2) (Pan/99) whole virus.

## 3 RESULTS

Of the 80 persons completing testing, 63 (79%) were exposed to poultry or their contaminated environment; 17 (21%) had office duties only. Exposure to potentially infected birds or virus‐contaminated environments occurred through outbreak control activities including collection of swabs from dead birds and poultry facilities, disposal of dead birds, incineration of birds, disinfection of equipment, and laboratory testing of poultry specimens. Many study participants (41%) reported >1 influenza‐like illness (ILI) symptoms and 18% reported ocular symptoms during their deployment with VAITF. The use of personal protective equipment varied by activity (Table 1).

**Table 1:** Characteristics of study participants, their exposures, use of personal protective equipment, and antibody test result

Characteristic	Result
Age (n = 80, median)	45 (range 24‐70)
Days to serum collection^a^ (n = 80, median)	21 (range 14‐228^b^)
Control activities (n = 80)	
Touch birds (%)	52 (65)
<1M from birds, no touching (%)	5 (6)
Incineration, no touching (%)	6 (8)
Office duties (%)	17 (21)
Influenza‐like illness symptoms^c^ (%)	
Feverishness (n = 80)	14 (18)
*T* > 37.8°C (n = 80)	3 (4)
Cough (n = 65)	20 (31)
Sore throat (n = 65)	21 (32)
Runny nose (n = 61)	13 (21)
Headache (n = 61)	9 (15)
Body ache (n = 61)	13 (21)
Red or watery eyes (n = 61)	11 (18)
Use of personal protective equipment	
Gloves (always wore during activity) (%)	
Touched dead birds (n = 50)	44 (88)
Touched infected birds (n = 32)	27 (84)
Swabbed birds (n = 29)	26 (90)
Collected environmental swabs (n = 14)	14 (100)
Loaded culled birds on/off trucks (n = 39)	30 (77)
Mask (always wore during activity) (%)	
Touched dead birds (n = 50)	14 (28)
Touched infected birds (n = 32)	10 (31)
Swabbed birds (n = 29)	6 (21)
Collected environmental swabs (n = 14)	7 (50)
Loaded culled birds on/off trucks (n = 39)	16 (41)
Antibodies to influenza A(H7N2) (n = 80)	1

a Days from first day of involvement in effort to control outbreak to day of serum collection. Range for seronegative persons (n = 79) was 14‐70 d.

b Sample collected on day 228 represents the second serum sample collected from the H7 antibody‐positive individual.

c Denominators vary because not all participants responded to each question.

Serum from 1 study participant tested positive for antibody to Tky/VA virus, achieving titers of 1:80 by MN in several independent assays (Table 2); sera from the remaining 79 participants tested seronegative. The specificity of the neutralizing antibody from the seropositive individual was determined by adsorption of sera with purified whole H7N2 and H3N2 viruses. Adsorption with H7N2, but not H3N2, removed the neutralizing antibody from the sera (Table 2). This result was confirmed by immunoblot analysis (data not shown), fulfilling WHO criteria for the serological identification of humans infected with avian influenza subtypes.^8^ This person (age mid‐20s) reported that he worked disposing of infected birds during the response and used gloves always and dust mask most of the time. Prior to outbreak, he had hunted waterfowl, but had limited exposure to domestic poultry. He reported an illness with onset 10 days prior to study enrollment with feverishness, cough, sore throat, and headache. He sought medical care at a private facility 1 day after illness onset. Chest X‐ray showed a right middle lobe infiltrate, and a diagnosis of lower respiratory tract infection was given. No diagnostic specimens were collected for microbiologic testing. Outpatient treatment consisted of unspecified intramuscular and oral antibiotics. He recovered fully.

**Table 2:** Serological response to H7N2 virus in antibody‐positive study participant involved in outbreak control operations^a^

Adsorption treatment	Neutralizing antibody titer against Tk/VA/02 (H7N2) virus^b^		ELISA IgM titer against^c^ Tk/VA/02 (H7) HA	
	Sample 1	Sample 2	Sample 1	Sample 2
None	80	80	1600	100
RBC only	80	80	1600	100
Tk/VA/02 (H7N2)	<20	<20	100	<100
Syd/97 (H3N2)	160	160	1600	100

ELISA, enzyme‐linked immunosorbent assay.

a Serum samples were collected from the individual 21 d (Sample 1) and 228 d (Sample 2) after the first day of work with outbreak control operations.

b Titers represent the reciprocal of the highest dilution of serum exhibiting 50% neutralization of 100 tissue culture infectious doses of virus. Untreated serum Sample 1 had a neutralizing antibody titer of 1:80 in 4 independent MN assays and was positive for antibody to purified Tk/VA/02 H7 HA by Western blot.

c Serum samples were diluted serially fourfold from a starting dilution of 1:100 in the first well. Titers represent the reciprocal of the highest dilution of the test serum achieving a value greater than the mean plus 3 standard deviations of 3‐6 negative controls at equivalent dilution.

A second blood sample collected from the antibody‐positive participant 228 days after joining outbreak control efforts was also positive by MN for antibody to Tky/VA HA (Table 2). No banked serum collected before participation was available for comparison. The 2 blood samples collected 21 days (10 days after symptom onset) and 228 days after beginning work on outbreak control efforts were tested for IgM antibodies to Tky/VA HA by ELISA. (Table 2) IgM antibody to Tky/VA HA was detected in the acute blood sample, but not in the convalescent blood sample. Adsorption of the acute blood sample with Tky/VA (H7), but not with Syd/97 (H3), removed the IgM antibody response suggesting that the antibody response was specific to Tky/VA (H7). Overall, these results suggest that a human infection with influenza A (H7) was temporally associated with a lower respiratory tract illness that occurred during participation in LP H7N2 avian influenza outbreak control efforts.

## 4 DISCUSSION

While this investigation was carried out in 2002, this report provides evidence of risk of human infection with a North American lineage LP H7N2 virus among persons exposed during control efforts. Exposure to and infection with an H7 influenza virus before participation in outbreak control efforts cannot be definitively excluded as no blood sample was available from the worker before engaging in VAITF activities. However, the presence of neutralizing antibodies and H7‐specific IgM antibodies in the acute blood sample collected 21 days after beginning disposal of infected carcasses and the absence of H7‐specific IgM antibodies in a convalescent blood sample 7 months later is consistent with an acute infection that likely occurred through exposure during the outbreak. While other etiologies for the pneumonia cannot be excluded, the temporal association of the pneumonia with exposure to H7 outbreak viruses and serologic evidence of acute infection are consistent with a respiratory illness resulting from avian influenza A(H7N2) infection.

While the precise route of transmission is not known, the H7‐infected worker had extensive exposure to infected birds during a 21‐day VAITF deployment through disposal activities that required a high level of direct contact with infected poultry. Current recommendations by the Centers for Disease Control and Prevention and the US Department of Agriculture for the use of personal protective equipment recommendations for workers engaged in control efforts (made after the Virginia outbreak) include protective goggles and disposable particulate respirators, in addition to disposable gloves, coveralls, and shoe covers.^9^ While the worker reported always wearing gloves, protective suit, and boots, he used a dust mask inconsistently and never used eye protection.

Serologic testing of human sera for antibodies to H7 viruses had not been optimized or standardized, and no human positive controls were available for comparison at the time of this study, and the study protocol did not include collection of respiratory samples, and the H7 antibody‐positive individual's illness was not identified until after his enrollment. Without a pre‐outbreak comparison of blood sample from this person, infection prior to working with the VAITF cannot be excluded; however, IgM results support our conclusions that this infection was associated with the VAITF deployment. Subsequent serologic investigations of a culture‐confirmed A(H7N2) human infection in 2003 yielded a convalescent neutralizing serum titer of 80, similar to the convalescent titer detected in the infected participant in the present study.^10^

The findings from this study further confirm that the North American lineage of LPAI A(H7N2) viruses that intermittently cause outbreaks among poultry can infect humans and cause respiratory illness; however, the overall risk of infection is low. While this study does not evaluate the effectiveness of personal protective equipment, its consistent use by persons engaged in control efforts should be employed as part of efforts to reduce the risk of infection. During future outbreaks among poultry, persons assisting with depopulation and surveillance of poultry, farmers, and poultry workers should be of high priority for illness monitoring and testing for infection with avian influenza viruses.
